# Development of New Multilocus Variable Number of Tandem Repeat Analysis (MLVA) for *Listeria innocua* and Its Application in a Food Processing Plant

**DOI:** 10.1371/journal.pone.0105803

**Published:** 2014-09-08

**Authors:** Hajime Takahashi, Chihiro Ohshima, Miku Nakagawa, Krittaporn Thanatsang, Chirapiphat Phraephaisarn, Yuphakhun Chaturongkasumrit, Suwimon Keeratipibul, Takashi Kuda, Bon Kimura

**Affiliations:** 1 Department of Food Science and Technology, Faculty of Marine Science, Tokyo University of Marine Science and Technology, Tokyo, Japan; 2 Department of Food Technology, Faculty of Science, Chulalongkorn University, Bangkok, Thailand; 3 Program in Biotechnology, Faculty of Science, Chulalongkorn University, Bangkok, Thailand; Cornell University, United States of America

## Abstract

*Listeria innocua* is an important hygiene indicator bacterium in food industries because it behaves similar to *Listeria monocytogenes*, which is pathogenic to humans. PFGE is often used to characterize bacterial strains and to track contamination source. However, because PFGE is an expensive, complicated, time-consuming protocol, and poses difficulty in data sharing, development of a new typing method is necessary. MLVA is a technique that identifies bacterial strains on the basis of the number of tandem repeats present in the genome varies depending on the strains. MLVA has gained attention due to its high reproducibility and ease of data sharing. In this study, we developed a MLVA protocol to assess *L. innocua* and evaluated it by tracking the contamination source of *L. innocua* in an actual food manufacturing factory by typing the bacterial strains isolated from the factory. Three VNTR regions of the *L. innocua* genome were chosen for use in the MLVA. The number of repeat units in each VNTR region was calculated based on the results of PCR product analysis using capillary electrophoresis (CE). The calculated number of repetitions was compared with the results of the gene sequence analysis to demonstrate the accuracy of the CE repeat number analysis. The developed technique was evaluated using 60 *L. innocua* strains isolated from a food factory. These 60 strains were classified into 11 patterns using MLVA. Many of the strains were classified into ST-6, revealing that this MLVA strain type can contaminate each manufacturing process in the factory. The MLVA protocol developed in this study for *L. innocua* allowed rapid and easy analysis through the use of CE. This technique was found to be very useful in hygiene control in factories because it allowed us to track contamination sources and provided information regarding whether the bacteria were present in the factories.

## Introduction

*Listeria* is a genus of gram-positive, facultatively anaerobic, non-spore forming bacteria [1]. *Listeria* spp. have been isolated from a variety of environmental sources, such as soil [2], river water [3], farm environments [4], and animal feed [5]. Additionally, *Listeria* spp. have been isolated from some foods [6], [7], [8] and food processing environments [9]. Because *Listeria monocytogenes*, a member of *Listeria* spp., can cause serious listeriosis infections in humans and ruminants, this pathogen is of significant health concern. *L. ivanovii* can also cause listeriosis infections in ruminants but rarely in humans [10].

Human listeriosis results from ingestion of *L. monocytogenes* via contaminated foods. Therefore, the United States has a zero-tolerance policy for contamination in processed foods, with certain exceptions, and the contamination level of *L. monocytogenes* in processed foods is strictly regulated at <100 cfu/g in the EU [1], [11].

Although *Listeria* spp. other than *L. monocytogenes* and *L. ivanovii* are not pathogenic for humans and animals, their presence in food products is still unacceptable to both the food exporters and importers. Therefore, in food industry, other *Listeria* spp. remain a major concern [12].

*L. innocua* in particular is considered to be similarly distributed and behave similarly to *L. monocytogenes* [12], [13] and is the most frequently isolated species [12], according to a report, among *Listeria* spp. in food manufacturing sites. For this reason, establishment of a monitoring method and clarification of contamination sources and routes are required.

Pulsed-field gel electrophoresis (PFGE) is widely used to assess the distribution of bacteria in foods and food manufacturing sites. PFGE, considered the gold standard technique due to its high resolution, has obvious disadvantages, including a complicated protocol, skill requirement, and time-consuming analysis [14]. In addition, multilocus sequence typing (MLST) and multilocus variable number of tandem repeat analysis (MLVA) have been developed as typing methods that use DNA sequence analysis. MLVA is a technique that types bacterial strains by utilizing the fact that the number of repeat units in the variable number tandem repeat (VNTR) region varies depending on the strain. Comparison of the numbers of repeats in multiple VNTR regions allows highly specific strain classification [15]. Analytical methods using VNTR regions have been developed for various food microorganisms, such as *Listeria monocytogenes* [14], [15], [16], *Escherichia coli* [17], *Bacillus anthracis* [18], *Salmonella enterica* [19], and *Vibrio parahaemolyticus* [20], showing the efficacy of MLVA as a strain identification method.

In this study, we developed an MLVA method effective for distribution assessment and tracking the contamination sources of *L. innocua*. Capillary electrophoresis (CE), with a simpler protocol and analysis procedure compared to sequence analysis, was used for MLVA because it is readily applied in food manufacturing sites. Moreover, we evaluated this MLVA protocol by using *L. innocua* isolated from a food factory and elucidated the contamination route of *L. innocua* in the factory.

## Materials and Methods

### MLVA primer design

Whole genome data of the *Listeria innocua* CLIP11262 strain was obtained from GenBank. Targeted VNTR regions of the *Listeria innocua* CLIP11262 strain were listed by using the Tandem Repeat Finder program (http://tandem.bu.edu/trf/trf.html) (Benson, 1999). Primer sets for amplification of each VNTR region were determined with the Primer Express Software (Life Technologies, Foster City, CA). The primer sets were designed within 300 bp from the end of VNTR regions to minimize the length of flanking regions.

### DNA extraction

Eighteen strains of *L. innocua* stored in our laboratory were used to evaluate of the developed MLVA method (Table 1). Of these strains, 8 strains were isolated from swab samples of food processing plants, and 10 strains were isolated from the environment in Japan (GPS 41.820; 140.653, 41.883; 140.638, 41.882; 140.639, 41.984; 140.681, 41.954; 140.709, 41.826; 140.735, 41.806; 140.715, 41.809; 140.708, 42.126; 140.735, 42.257; 140.254). The sampling site is located in open access area and no specific permissions are required to collect samples.

**Table 1:** Characteristics of *L. innocua* isolates used for development of the MLVA method.

Strains	Sampling Site
1-2-1	Food processing plant
1-8-1	Food processing plant
1-25-1	Food processing plant
1-28-1	Food processing plant
1-29-1	Food processing plant
2-28-1	Food processing plant
2-29-1	Food processing plant
2-35-1	Food processing plant
6-5-3	Environment
7-4-1	Environment
6-9-2	Environment
7-10-3	Environment
11-10-1	Environment
6-12-1	Environment
6-16	Environment
7-15-2	Environment
26-1-1	Environment
57-2	Environment

Strains were grown in trypticase soy broth (TSB; Becton Dickinson and Company) overnight at 37°C. Bacterial cells were harvested from 1 mL TSB by centrifugation at 8,000×*g* for 3 min, and the supernatant was removed. DNA extraction was conducted using the NucleoSpin Tissue Kit (Macherey-Nagel GmbH & Co. KG, Düren, Germany), according to the manufacturer's protocol.

### Amplification of VNTRs

PCR was performed in a final volume of 50 µL. The PCR reaction mix contained 10 mM Tris-HCl (pH 8.3), 50 mM KCl, 1.5 mM MgCl_2_, 0.2 mM each of dNTPs, 1 mM forward primer, 1 mM reverse primer, 25 ng of template DNA, and 0.5 U Takara Taq DNA polymerase (Takara Bio). Amplification was performed using the GeneAmp PCR System 9700 Thermalcycler (Life Technologies). The following parameters were used for amplification: 94°C for 5 min, 30 cycles of 94°C for 30 s, 60°C for 40 s, and 72°C for 1 min, and 72°C for 1 min.

### Capillary electrophoresis

The sizes of PCR products were analyzed by capillary electrophoresis (QIAexcel, Qiagen) and determined by QIAxcel ScreenGel software (Qiagen). The DNA High Resolution Kit Gel Cartridge (Qiagen) was used in this study. The number of repeat units in each VNTR region were calculated from their product size using modeling sizes, which were defined by the sequence data of *Listeria innocua* CLIP 11262 strain. The sizes of PCR products obtained from capillary electrophoresis were assumed to be the closest modeling size of PCR products, and the number of repeat units were determined.

### Confirmation of the number of repeat units using sequencing

The PCR products obtained were purified using Agencourt AMPure (Beckman Coulter), according to the manufacturer's protocol. Following purification, the amplification products were sequenced using the BigDye Terminator v3.1 Cycle Sequencing Kit (Life Technologies), according to the manufacturer's protocol. The primer sets used for sequencing reactions were the same as those used for amplification. After completion of the sequencing reaction, the sequence was determined using an ABI 3130 Genetic Analyzer (Life Technologies). A consensus region was resolved by Genetix-ATGC using the sequences from each primer, and the numbers of repeat units were counted. The numbers of repeat units were compared with the results of capillary electrophoresis. Furthermore, the differences between sequences in repeat units were checked.

### Evaluation of the developed method using food industry strains

The developed method was evaluated by typing 60 *L. innocua* strains isolated from a food processing plant. These strains were obtained from laboratory collection which covers a two-year period of monthly environmental monitoring isolates from various processes, including steam, chilling, cutting, freezing, and packing. These environmental swab samples were intentionally taken from the areas that are difficult to access for cleaning in order to monitoring the persistent of *L. innocua*. Cultivation and extraction of DNA were conducted as previously described. VNTR regions of TR-D, TR-E, and TR-J were amplified using developed methods. PCR products were analyzed by capillary electrophoresis, and the numbers of repeat units were calculated. Allele numbers were assigned based on the numbers of repeat units, which were calculated from the results of capillary electrophoresis.

## Results

### Determination of VNTR regions

In total, 100 candidate VNTR regions were obtained as a result of the whole genome database search of the *L. innocua* CLIP11262 strain using Tandem Repeat Finder. Among them, three VNTR regions, TR-D, TR-E, and TR-J, with the sequence length suitable for MLVA using CE, were chosen, and their corresponding primer sets were designed (Table 2). Table 3 shows characteristics of the VNTR regions used in the MLVA. The repeat units in the three VNTR regions had 6–9 bases each (Table 3).

**Table 2:** Primer sets for MLVA analysis.

TR	Primer	Sequence (5′-3′)	Location in CLIP11262	Amplicon size
TR-D	D-forward	GACAAAAGTAAGTCATGCGGGTATTT	1124253–1124395	440 bp
	D-reverse	TAGCTACAATCGGATTAACGG		
TR-E	E-forward	GTACCTCCATTTGCTGTTCCA	1924244–1924320	256 bp
	E-reverse	ATGTTATCCACCTTCAAGTAACTG		
TR-J	J-forward	ATGTTTGTGTTCTCAGTTGCC	2741368–2741485	369 bp
	J-reverse	CTACCAAGGATTACTACAAGAAC		

**Table 3:** Sequence of repeat unit and part of the flanking region of the variable number of tandem repeats (VNTR) regions.

Locus	Flanking region (5′)		Sequence of repeat unit	Flanking region (3′)		Allele number
	Length(bp)	partial sequence		Length(bp)	partial sequence	
TR-D	161	agccactgttggagaagc	CGGTAGACC	117	caatcactccagtagacc	{X-(161+117)}/9
TR-E	58	tagtacctcctc	CATCGG	90	aatcaactaatg	{X-(58+90)}/6
TR-J	79	ctgctggttgaatcggat	TTACTGGGT	173	ctaccggaactactggat	{X-(79+173)}/9

Formula for calculating the allele number on the basis of the fragment size.

### Development of the MLVA method for *L. innocua*

The 18 *L. innocua* strains stored in our laboratory derived from food factories and the environment were each subjected to MLVA. In this study, the numbers of repeat units were calculated by using the model size designed first after determining the length of the PCR product using CE. As a result, the numbers of repeats in the VNTR regions were 9–21, 13–18, and 3–14 in TR-D, TR-E, and TR-J, respectively. The numbers of alleles in the regions were 6, 5, and 7 in TR-D, TR-E, and TR-J, respectively.

Next, the numbers of repeat units obtained from the CE result were confirmed by sequencing. The following concept was applied to counting the number of repeats by sequencing. Mutation of a base per three bases in a repeat unit was allowed. For example, for the repeat unit in TR-E, CATCGG, if it was mutated to CTTCAG or CATCCG, it was also treated as a repeat unit.

Consequently, TR-D in 16 out of 18 strains was found to have different bases equivalent to the two repeats, which were 12 bases (Table 4). In addition, there were two strains in which the number of repeat in TR-J was different between the CE results and sequencing. The sequencing results confirmed a slight difference in the sequence of the repeat unit in all three regions.

**Table 4:** Compared MLVA profile of 18 *L. innocua* strains which identified by sequencing or capillary electrophoresis.

	D						E					J				
Strain no.	Ampliconsize (bp)	Closest amplicon size of the RU model	Number of repetitions from CE	Number of repetitions from sequencing	Number of the difference of repetitions	Number of inserted sequence (bp)	Ampliconsize (bp)	Closest amplicon size of the RU model	Number of repetition from CE	Number of repetition from sequencing	Number of the difference of repetition	Ampliconsize (bp)	Closest amplicon size of the RU model	Number of repetition from CE	Number of repetition from Sequencing	Number of the difference of repetition
1-2-1	479	476	22	20	2	12	243	244	16	16	0	382	378	14	14	0
1-8-1	478	476	22	20	2	12	244	244	16	16	0	373	369	13	13	0
1-25-1	479	476	22	20	2	12	244	244	16	16	0	373	369	13	13	0
1-28-1	478	476	22	20	2	12	244	244	16	16	0	374	369	13	13	0
1-29-1	480	476	22	20	2	12	246	244	16	16	0	347	351	11	10	1
2-28-1	487	485	23	21	2	12	244	244	16	16	0	346	342	10	10	0
2-29-1	478	476	22	20	2	12	245	244	16	16	0	373	369	13	13	0
2-35-1	480	476	22	20	2	12	245	244	16	16	0	346	351	11	11	0
6-5-3	397	395	13	11	2	12	226	226	13	13	0	307	306	6	6	0
7-4-1	381	381	11	9	2	12	232	232	14	14	0	301	297	5	5	0
6-9-2	369	368	10	10	0	0	257	256	18	18	0	380	378	14	14	0
7-10-3	388	386	12	10	2	12	226	226	13	13	0	310	306	6	6	0
11-10-1	374	377	11	9	2	12	234	232	14	14	0	301	297	5	5	0
6-12-1	369	368	10	10	0	0	257	256	18	18	0	380	378	14	14	0
6-16	387	386	12	10	2	12	256	256	18	18	0	338	342	10	9	1
7-15-2	454	458	20	18	2	12	243	244	16	16	0	283	279	3	3	0
26-1-1	387	386	12	10	2	12	226	226	13	13	0	308	306	6	6	0
57-2	377	377	11	9	2	12	237	238	15	15	0	301	297	5	5	0

Sequence type (ST) was determined in each strain based on the pattern of the repeat numbers in TR-D, TR-E, and TR-J. When CE was used, the 20 strains were classified into 12 types, as opposed to 11 types when using sequence analysis.

### Evaluation of the established MLVA protocol using strains isolated from a food factory

Sixty *L. innocua* strains that were isolated from a food factory were subjected to MLVA using CE. As a result, the strains were classified into 5 patterns for TR-D and 4 patterns for TR-E and TR-J, allowing us to classify the strains into 11 patterns by combining the three regions (Table 5).

**Table 5:** The result of MLVA analysis for 60 *L. innocua* isolated from food processing plant.

Sample no.	D	E	J	MLVA-ST	Source of isolate
	No. of repeat unit	No. of repeat unit	No. of repeat unit		
124	12	13	6	1	Packaging area
133	12	13	6	1	Chilling area
235	12	13	6	1	Worker's hand
276	19	16	11	2	Worker's boots
13	20	16	11	3	Chilling area
9	20	17	11	4	Freezing area
277	23	15	13	5	Freezing area
77	23	16	11	6	Cutting area
20	23	16	11	6	Cutting area
29	23	16	11	6	Cutting area
260	23	16	11	6	Heating area
262	23	16	11	6	Heating area
48	23	16	11	6	Packaging area
4	23	16	11	6	Cutting area
24	23	16	11	6	Worker's hand
25	23	16	11	6	Freezing area
26	23	16	11	6	Freezing area
33	23	16	11	6	Cutting area
43	23	16	11	6	Freezing area
54	23	16	11	6	Chilling area
75	23	16	11	6	Packaging area
84	23	16	11	6	Packaging area
87	23	16	11	6	Freezing area
98	23	16	11	6	Freezing area
107	23	16	11	6	Packaging area
108	23	16	11	6	Chilling area
121	23	16	11	6	Cutting area
143	23	16	11	6	Cutting area
148	23	16	11	6	Cutting area
175	23	16	11	6	Cutting area
185	23	16	11	6	Freezing area
211	23	16	11	6	Chilling area
252	23	16	11	6	Packaging area
290	23	16	11	6	Heating area
299	23	16	11	6	Cutting area
31	23	16	11	6	Packaging area
82	23	16	11	6	Packaging area
117	23	16	11	6	Heating area
126	23	16	11	6	Chilling area
303	23	16	11	6	Cutting area
144	23	16	11	6	Cutting area
281	23	16	11	6	Cutting area
100	23	16	12	7	Cutting area
40	23	16	13	8	Heating area
78	23	16	13	8	Worker's hand
152	23	16	13	8	Heating area
181	23	16	13	8	Cutting area
187	23	16	13	8	Freezing area
308	23	16	13	8	Heating area
30	23	16	13	8	Heating area
304	23	16	13	8	Chilling area
286	23	16	13	8	Cutting area
306	23	16	13	8	Freezing area
269	23	16	14	9	Freezing area
1	23	17	10	10	Freezing area
3	23	17	11	11	Packaging area
5	23	17	11	11	Cutting area
311	23	17	11	11	Chilling area
313	23	17	11	11	Cutting area
49	24	16	11	11	Chilling area

When focusing on the distribution of the MLVA patterns in the factory, *L. innocua* isolated from areas for freezing/packaging showed six MLVA patterns, the highest number of patterns when compared to other areas. The strains classified as MLVA-ST6 were isolated from heating, freezing, and packaging process areas in the factory.

## Discussion

*L. monocytogenes*, which is pathogenic to humans, is strictly controlled for in food factories, and a number of methods for tracking contamination sources of this pathogen have been published [21], [22]. In this study, we developed an MLVA method for *L. innocua*, which is predicted to be an indicator bacterium of *L. monocytogenes* contamination due to its similar behavior [12], [13], and tracked the contamination source using strains isolated from an actual food factory.

DNA typing methods that are widely used for epidemiological surveys and tracking contamination sources include PFGE [22]. However, the disadvantages of PFGE range from a complicated protocols, time consumption, to difficulty in data comparison due the results being a band pattern [1]. One DNA typing method reported for *L. innocua* is random amplified polymorphic DNA (RAPD) [12]. RAPD has low reproducibility, and the analysis is cumbersome to share because the electrophoresis results have to be converted into comparable data. On the other hand, in MLVA, the protocol is easier, and the analysis is shorter than that of PFGE and RAPD. In addition, because the data obtained are not a band pattern, it is easy to share and compare the data [1]. Because of those properties, MLVA is very convenient for food companies and other industries with multiple factories.

In the MLVA protocol established in this experiment, the analytical conditions allowed for base mismatching per three bases (3/9, 2/6, and 3/9 bases in TR-D, TR-E, and TR-J regions, respectively) included in the repeat unit. The length of flanking regions outside the repeat units was fixed in the model case of each region [23]. When the typing profiles from the analysis under these conditions were compared with that from the sequence analysis, the number of the repeat unit was same between CE and sequencing in TR-E. There were two strains in which the length of the PCR product from TR-J analyzed by CE was 4 bases longer than that determined by sequencing. However, other strains had the same number of repeats between the two methods. Sixteen out of 18 strains had different numbers of repeat units in TR-D between CE and sequencing. When the sequences were compared between the CLIP11262 strain used for setting conditions and 18 strains used in the experiment, insertions of 12 bases in the flanking regions were found in strains with different numbers of repeat units. Because the repeat unit in TR-D has 6 bases, insertion of these 12 bases led to an estimated two more repeats of the unit. These results revealed that CE (QIAexcel) used in this experiment was sufficiently specific and usable in the analysis of the numbers of repeat units in MLVA.

Analysis of *L. innocua* isolated from food processing factory using MLVA-CE established in this study resulted in the classification of 60 strains into 11 STs and revealed the presence of *L. innocua* with multiple STs in this factory. Many of the 60 strains isolated from the factory were classified as ST6. The strains classified as ST6 were isolated from many areas of factory during different time period of sampling, revealing that this strain of *L. innocua* is widely distributed in the factory and transferred from area to area. In this factory, the area for processing chicken meat before heating and the area for handling products after steaming are connected by steaming aperture, but the workers are completely independent between the areas. During operation of steamer, there is no situation in which *L. innocua* attached to the raw materials constitutively enters the post-steaming area. However, when cleaning procedure is applied after the end of the operation, it is likely that the organism survived in pre-heating area could pass through post-steaming area by this channel and disperse to the areas of cooling, cutting, freezing, and packing by various routes. Due to the fact that *L. innocua* ST6 was isolated from the processing areas at all two-year period, it is likely that *L. innocua* resides and persistently contaminate in the factory.

Although this factory was routinely cleaned carefully and the air temperature was maintained at 10°C, ST6 strains were shown to reside in many areas. With this in mind, these strains are likely to be highly resistant to various environmental stresses. Other strains were likely to reside at lower frequency than that of ST6, as they were isolated from two sites at most.

In this study, we established an MLVA protocol for *L. innocua* by using CE that allows a simpler work process, shorter analysis duration, and cost reduction. In the evaluation of the established technique using strains isolated from a food factory, this MLVA protocol allowed us to obtain information on the actual status of contamination and bacterial is residence in the factory, demonstrating the high usefulness for hygiene control in factories. These data indicate that the MLVA-CE protocol is very effective as a highly identifiable and simple typing method.
